# Large-scale *Anopheles arabiensis* egg quantification methods for mass-rearing operations

**DOI:** 10.1186/s12936-016-1119-7

**Published:** 2016-02-06

**Authors:** Hamidou Maïga, David Damiens, Abdoulaye Diabaté, Roch K. Dabiré, Georges A. Ouédraogo, Rosemary S. Lees, Jeremie R. L. Gilles

**Affiliations:** Institut de Recherche en Sciences de la Santé/Centre Muraz, BP 390, Bobo-Dioulasso, Burkina Faso; Insect Pest Control Laboratory, Joint FAO/IAEA Division of Nuclear Techniques in Food and Agriculture, International Atomic Energy Agency, Wagramerstraße 5, PO Box 100, 1400 Vienna, Austria; Université Polytechnique de Bobo-Dioulasso, 01 BP 1091, Bobo-01, Burkina Faso

**Keywords:** *Anopheles arabiensis*, Egg weight, Dried eggs, Mass-rearing, Sterile insect technique

## Abstract

**Background:**

The success of the sterile insect technique relies, among other things, on the continuous release of over flooding numbers of sexually competitive sterile males into the target area. To produce sufficiently large quantities of sterile males, rearing protocols need to be optimized including the development and validation of a standardized egg quantification method.

**Methods:**

Batches of 1000 freshly laid eggs collected from standard rearing cages were counted, gently dried under laboratory conditions (27 ± 1 °C, 75 ± 5 % RH) and combined so that 1000–8000 eggs were weighed, to calculate the correlation between weight and number. The actual counted egg number and the egg number estimated by weighing were further compared for samples of 1000, 3000 and 4000 eggs collected from both standard and mass-rearing cages. The effect of drying, brushing and weighing on egg hatch rate was evaluated in three samples each of 1000 fresh and 1000 dried eggs, and in batches of 1000, 3000 and 4000 dried eggs. Pupal production and adult life history traits were assessed for dried eggs hatched and reared in mass-rearing trays. Expected egg numbers and actual observed mean egg numbers were compared after gentle drying, and after applying a rapid drying method exposure to wind speed of 1.8 m/s for 30 min.

**Results:**

A significant positive relationship between the number of dried eggs and egg weight was observed and the equation ‘Weight (mg) = (0.00399 × Number of counted eggs) + 0.536 was derived. The actual counted mean egg number and the egg number estimated by weighing were similar for samples from small rearing cages but significantly lower for samples of 3000 and 4000 egg samples collected from mass-rearing cages. No negative effect of the drying, brushing and weighing process on egg hatch rate was observed. No significant difference was observed in any life history trait between adults reared from dried or from fresh eggs up to twenty-one days post emergence. The mean number of eggs counted from a given replicate’s weight was significantly higher for egg batches fast dried with a suction device compared to those dried with a gentle drying method (fast: 1075 ± 9, gentle: 1024 ± 7).

**Conclusion:**

An equation has been derived to allow accurate quantification of dried *Anopheles arabiensis* eggs based on weight, enabling more accurate quantification of eggs for consistent larval rearing density to be achieved. Eggs can be dried for weighing in a manner which does not impair the quality of resulting adults.

## Background

The sterile insect technique (SIT) is a species-specific and environmentally-friendly method of insect pest control based on the release of large numbers of sterile insects, usually males [1]. Sterilized by exposure to gamma- or X-rays before release, the laboratory-reared males will transfer their sterile sperm to wild females during mating, causing early embryonic death and a progressive decrease in the pest population. The success of the SIT depends mainly on a continuous release of high numbers of sterile males which need to be sexually competitive with wild males within the target area [2]. To produce sufficient numbers, mass-rearing equipment and optimized rearing protocols have to be developed [3]. The insect pest control laboratory (IPCL) of the Joint FAO/IAEA Division of Nuclear Techniques in Food and Agriculture has been supporting studies to assess the feasibility of integrating the SIT into programmes for area wide control of the malaria vector *Anopheles arabiensis* and of the Dengue/Chikungunya/Zika vectors *Aedes aegypti* and *Aedes albopictus* in different countries. The IPCL has developed mass-rearing equipment and suitable rearing methods such as a larval mass-rearing unit [4], a larvae-pupae separator [5] and adult mass-rearing cages (MRC) [6]. These tools are now available for use by projects subject to validation and optimization under operational conditions.

In mosquito mass-rearing, the management and quantification of large quantities of eggs produced in MRC is critical. Various standardized methods have already been developed for quantifying egg production in mosquito mass-rearing settings using volumetric estimation [7–10] and digital image analysis [11, 12]. Manually counting eggs is time consuming, could easily lead to errors [13], and is highly impractical in a mass-rearing setting. Rapid estimation of egg number based on image analysis of photographed egg papers is possible [12, 13] but does not allow for easy distribution of the quantified eggs for larval rearing.

To manipulate and weigh eggs they need to be dried, since handling and quantification of fresh eggs is impractical due to their stickiness and tendency to clump [9]. In *Anopheles albimanus* [14], *Anopheles quadrimaculatus* [8], *Anopheles stephensi* [15] and recently *Anopheles arabiensis* [9], it has been shown that drying eggs for a short period has no effect on egg hatch rate. However, the effect of drying eggs on subsequent life history traits that could influence male competitiveness has not been studied. Damage caused by egg manipulation may not be obvious from the hatch rate, but there may be more subtle effects on the individual adults hatching from the eggs.

The aim of the present study was to develop and validate a standardized method to quantify batches of eggs, collected from routine insectary-scale rearing. Such a method would allow rearing trays to be filled with an equal number of eggs, leading, if fed with the same quantity of larval food, to a synchronized larval development. It has been shown that while overfeeding can increase larval mortality, overcrowding can seriously impede larval development resulting in production of smaller adults [10 and references therein]. The method was then adapted to, and validated for mass-rearing settings. For this the main challenge was to overcome the challenge presented by the debris, mainly parts of dead adults, which is present in the slurry of eggs collected from the mass-rearing cages. This study also aimed to assess the effect of drying and weighing on pupal production, emergence rate, adult body size, adult longevity, male mating ability, and female fecundity. The effect of a rapid drying method on the hatch rate and accuracy of egg number estimation was also assessed and results compared to those obtained using the gentle drying method.

## Methods

### Rearing and egg collection

The Dongola strain of *An. arabiensis*, originating from Sudan, was maintained at the IPCL (Seibersdorf, Austria) in small rearing cages (30 × 30 × 30 cm BugDorm Model 1, Taiwan) following the rearing procedure described by Damiens et al. [16]. Eggs were collected in black-lined plastic cups containing a wet sponge covered by a filter paper which was collected from the cages and replaced twice a week.

*An. arabiensis* were also reared in a modified version of the mass-rearing cages (MRC) described by Balestrino et al. [6]. Between 5000 and 10000 adults were offered defrosted bovine blood in the Hemotek membrane feeder (Discovery Workshops, UK) [17] for one to 2 h twice per week. Sugar solution (5 %) was available ad libitum. In the MRC, females laid eggs directly onto water that was flushed out of the MRCs every day to collect eggs [6] for use in the experiments. Eggs were collected from the MRC through three stacked sieves (mesh sizes 900, 300 and 50 µm). The 900 µm and 300 µm sieves retained the dead mosquito bodies and body parts such as legs and wings, and finally the eggs were collected in the 50 µm sieve.

Eggs collected for use in the experiments were either gently allowed to dry on paper in the rearing room until dry enough to brush from the paper, which took a period of 3–5 h, or rapidly dried using a suction device with a suction device set to 1.8 m/s for 30 min.

### Weight estimation of dried eggs

Eight batches of 1000 freshly laid eggs were counted and allowed to dry under laboratory conditions (27 ± 1 ℃, 75 ± 5 % RH) on a paper towel in a small tray (30 × 40 cm) for 3–5 h. Dried eggs were gently brushed onto tracing paper (Duerer-Hase, A4, 25 sheet, 80 g, article number 101600, LIBRO, Austria) then poured into 200 µl Eppendorf tubes (Hamburg, Germany). One clean Eppendorf tube was used to weigh each sample of 1000 eggs. The eggs were then transferred into another tube to weigh the cumulative amounts (2000, 3000, 4000, 5000, 6000, 7000 and 8000 eggs). Five replicates of each egg number were weighed from different egg batches collected from small rearing cages on consecutive weeks.

### Validation of method for egg number estimation

**Fig. 1:**
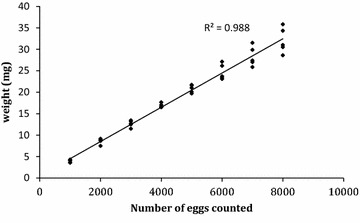
Correlation between weight and counted number of dried *Anopheles arabiensis* eggs, counted by stereomicroscope and then weighed. Data is presented from five replicates each of 1000, 2000, 3000, 4000, 5000, 6000, 7000 and 8000 dried eggs

The efficacy of weighing dried eggs for estimating absolute numbers from small rearing cages was validated by weighing out egg samples of 1000, 3000 and 4000 based on the previously measured correlation between egg number and weight (Fig. 1). Each sample was then counted and the observed mean number compared to the expected number of eggs. A total of fourteen samples of 1000, six of 3000 and six of 4000 eggs were used for this validation.

### Effect of drying, brushing and weighing on egg hatch rate

Six samples of 1000 freshly laid eggs were counted under a stereomicroscope and allowed to hatch immediately in small trays (30 × 40 cm) with 1.5 L of deionized water and 4 ml of 1 % IAEA larval food. A further six samples each of 1000, 3000 and 4000 eggs were dried over 3–5 h, brushed and weighed before being hatched in the same way. Egg hatch was monitored once daily for 3 days: all first-instar larvae were removed, counted and the mean egg hatch rate compared between samples of 1000 fresh and dried eggs, and between dried samples of 1000, 3000 and 4000 eggs.

### Effect of egg number estimation on life history traits

The following life history traits were assessed and compared between pupae and adults obtained from fresh (control) and dried eggs: pupation rate, pupal emergence rate, adult longevity, male mating ability, female fecundity and adult body size.

For the control, eggs collected on filter papers were counted under a stereomicroscope, and three samples of 4000 eggs were dispensed into three larval mass-rearing trays (100 × 60 × 3 cm) [4]. For the treatment eggs (dried, brushed, and weighed), the egg papers were dried for 3–5 h and three samples of 4000 dried eggs were subsequently dispensed into three larval mass-rearing trays. The room temperature was kept at 30 °C leading to a water temperature of 28 °C in the rearing trays. The IAEA larval diet [18] was dispensed every day until pupation as follows: 100 ml/tray for the five first days, 150 ml between the 6th and the 8th and 100 ml on the 9th day. All pupae were picked using a micropastette and counted to determine the total number of pupae per tray. Pupation rate was calculated from the total number of pupae divided by the number of eggs added to the tray. Of the pupae collected, fifty male and female pupae from each tray, collected on the same day, were placed in separate cages in order to count the number of adults that emerged. Six replicates were carried out for treatment and control: dried or fresh eggs.

The longevity of fifty newly emerged males and fifty females was assessed in small rearing cages (30 × 30 × 30 cm). Adults were supplied with 5 % sugar water ad libitum. Dead mosquitoes were removed every day, counted and separated by sex until the last individuals died. Three replicates were done for each group of mosquitoes collected either from fresh or dried eggs.

Male mating ability and female fecundity were assessed with fifty males (either from dried or fresh eggs) and fifty females obtained from fresh eggs placed together in small rearing cages (30 × 30 × 30 cm BugDorm Model 1, Taiwan). Females were offered a blood meal with the Hemotek membrane feeder [16] for 1 h on the 5th and 6th days. All blood fed females were then kept for 3 days for egged *en masse* in plastic cups containing a filter paper on wet sponge. Collected eggs were counted under a stereomicroscope to assess the fecundity of females from dried and from fresh eggs. To assess the insemination status of females, surviving females were dissected after the oviposition period under a stereomicroscope and the presence/absence of spermatozoa in the spermathecae was observed under a compound light microscope at 400× magnification.

The wing lengths were measured from thirty male and twenty-one female mosquitoes obtained from each treatment, dried and fresh eggs [19].

### Effect of a rapid drying method on egg number estimation and hatch rate

For all previous experiments, eggs were dried gently at room temperature over a period of 3–5 h. This method takes time and may not be practical for mass-rearing. A faster method requiring only 30 min, using a suction device with a fan set to 1.8 m/s was developed by Dame et al. [7] and recently validated by Khan et al. [9].

For each treatment, dried eggs were brushed from the paper on which they were laid and eggs from several papers pooled. From the pool, seven samples of 1000 eggs, as estimated by weight, were collected. After weighing, the number of eggs was counted under a stereomicroscope. A comparison was made between expected egg number and actual observed egg number after gentle drying and after rapid drying.

For each drying protocol, seven samples of around eighty eggs were taken from the pool of eggs, placed into small hatching cups (5 cm in diameter) containing deionized water with 1 ml of 1 % IAEA larval food. The hatch rates were compared after 3 days of hatching by counting the hatched and unhatched eggs.

### Adaptation of egg number estimation method to a mass-rearing scale

Because of differences in the egg collection process between small rearing cages (where eggs are laid directly onto filter papers) and mass-rearing cages (laid on water, flushed out and filtered through sieves), the effect of collection from mass-rearing cages on dried egg weight and hatch rate was assessed.

The efficiency of the dried egg weight estimated for eggs collected from mass-rearing cages was validated by comparing the expected number of eggs after estimation by weighing and the actual counted number of eggs. Eggs collected from the mass-rearing cages were dried and weighed out into batches expected to comprise 1000, 3000 or 4000 eggs, based on previous quantification experiments conducted with eggs collected from small rearing cages (Fig. 1). Six batches of each size were weighed out. The number of eggs in each batch was then counted under a stereomicroscope to obtain the actual observed number.

Three samples of 1000 eggs collected from the mass-rearing cages and gently dried were rinsed into three small trays (30 × 40 cm) with 1.5 L of deionized water and 4 ml of 1 % IAEA larval food and the hatch rate assessed by harvesting all first instar larvae. Hatch rate was compared to that of three samples of 1000 eggs freshly collected from small rearing cages and rinsed directly into trays (control).

### Statistical analysis

All statistical analyses were performed using Graph Pad software. A linear regression was performed between estimated egg weight and the actual number of eggs. Pearson correlation coefficient was calculated and tested for each relationship between estimated weight and counted number of eggs. 1-way Analysis of Variance (ANOVA) and Paired *t* test comparison were used to compare all mean egg hatch rates when egg hatch proportions were arcsine-square-root transformed using Microsoft Excel 2007 (Microsoft, WA, USA). Egg data were checked for normality with the Shapiro–Wilk test and a Chi square test performed in order to compare expected number of eggs with mean observed numbers from both small cages and mass-rearing cages. Wing length was measured, tested for normality with the Shapiro–Wilk test and mean length of male and female mosquitoes obtained from either fresh or dried eggs compared using the Student’s *t* test. Adult longevity was assessed using the Kaplan–Meier estimator with the log-rank test to determine significance.

## Results

### Weight estimation

The relationship between the number of eggs counted and their weight is shown in Fig. 1. The Pearson correlation coefficient between both parameters was 0.988, a highly significant (*P* < 0.0001) positive relationship. The equation describing the linear regression was weight (mg) = (0.00399 × number of counted eggs) + 0.536.

### Validation of method for egg number estimation

No significant difference was observed when the mean numbers of eggs calculated after weighing samples of 1000 (χ^2^ = 11.70, df = 13, *P* = 0.5), 3000 (χ^2^ = 7.497, df = 5, *P* = 0.1) or 4000 (χ^2^ = 3.567, df = 5, *P* = 0.6) eggs were compared to the expected value (Table 1).

**Table 1:** Comparison between mean absolute numbers of *Anopheles arabiensis* eggs, collected from either small rearing cages (SRCs) or mass-rearing cages (MRCs) and counted using a stereomicroscope, and the number of eggs expected based on previously measured correlation between egg number and weight

Number of eggs expected	Number of eggs counted (mean ± SE (N))			
	SRCs	*P*	MRCs	*P*
1000	989 ± 11 (14)	ns	896 ± 23 (6)	ns
3000	3032 ± 39 (6)	ns	2557 ± 80 (6)	*
4000	4270 ± 32 (6)	ns	3737 ± 137 (6)	*

A Chi square test was performed for all pairs of egg numbers except between cage type (SRCs and MRCs)

* Significant difference with *P* < 0.0001, *ns* no significant difference (*P* > 0.05). *se* standard error and (*N*) number of assessed samples of eggs

### Effect of drying, brushing and weighing on egg hatch rate

No significant differences were observed when the hatch rate of batches of 1000 fresh control eggs was compared to that of 1000 eggs estimated by weight (fresh: 66.2 ± 11.8 %, dried: 67.5 ± 4.1 %: t = 0.018, df = 5, *P* = 0.9). However, when the hatch rate of the eggs estimated by weight (67.5 ± 4.1 %, 75.2 ± 2.4, 73.6 ± 2.2 % for 1000, 3000 and 4000 eggs, respectively) was analysed, a significant difference (ANOVA, F_2,15_ = 12.11, *P* = 0.0007) though no relationship with batch size was observed.

### Effect of egg number estimation on life history traits

**Fig. 2:**
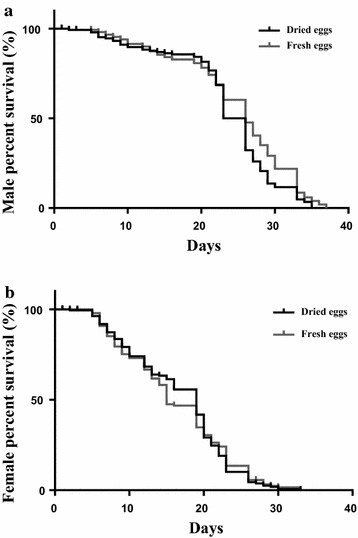
Survival curves estimated using the Kaplan–Meier method of **a** mated males and **b** mated females, reared from eggs which were either hatched immediately after collection (fresh) or dried and quantified before hatching (dried)

No significant difference was observed for pupation rate, pupal emergence rate, adult longevity, male mating ability, female fecundity or adult body size between individuals resulting from dried and fresh eggs (Table 2). A Log-rank (Mantel-Cox) test found a greater longevity in male mosquitoes reared from fresh egg batches (χ^2^ = 7.061, df = 1, *P* = 0.007); the last mosquito died on day 35. However, no statistical difference was found when survival was monitored for the first twenty-one days post-emergence (χ^2^ = 0.004, df = 1, *P* = 0.9) (Fig. 2a). The longevity was similar in females obtained from fresh and dried eggs (χ^2^ = 0.008, df = 1, *P* = 0.9) (Fig. 2b).

**Table 2:** Effect of drying, brushing and quantifying *Anopheles arabiensis* eggs on adult life history traits in resulting mosquitoes

Parameters	Egg type		
	Fresh	Dried	
PR (%)	58.5 ± 3.2	63.1 ± 10.3	t = 0.513, df = 2, *P* = 0.6
ER (%)	94.6 ± 1.4	93.4 ± 1.5	t = 0.736, df = 5, *P* = 0.4
IR (%)	74.2 ± 8.2	84.6 ± 7.2	t = 1.704, df = 2, *P* = 0.2
F	772.3 ± 118.9	619.3 ± 331.4	t = 0.671, df = 5, *P* = 0.5
WL male (µm)	3122.8 ± 23.1	3118.5 ± 16.1	t = 0.132, df = 29, *P* = 0.8
WL female (µm)	3433.05 ± 22.4	3400.27 ± 18.4	t = 1.554, df = 20, *P* = 0.1

Eggs were collected from small rearing cages, and either hatched immediately or dried before hatching into larval mass-rearing trays (100 × 60 × 3 cm), and then reared to adulthood. Results of a t test comparison between fresh and dried egg batches are presented

Within each egg type all parameters were found not to be significantly different from each other (*t* test) (*P* > 0.05)

*PR* pupation rate as a proportion of eggs, *ER* emergence rate as a proportion of total pupae, *IR* insemination rate as a proportion of all female adults, *F* fecundity (mean number of eggs per treatment), *WL* wing length

### Effect of a rapid drying method on egg number estimation and hatch rate

The mean observed number of eggs counted from batches of the same estimated number was significantly higher in eggs dried for 30 min with a suction device compared to those left for 3–5 h to dry (rapid: 1075 ± 9, gentle: 1024 ± 7: t = 5.773, df = 4, *P* = 0.004). No significant difference in the mean hatch rate was observed between eggs subjected to the two drying methods (rapid: 88.4 ± 2.2 %, gentle: 78.7 ± 5 %: t = 1.385, df = 6, *P* = 0.2).

### Adaptation of egg number estimation method to a mass-rearing scale

The actual mean counted number of eggs from MRCs was significantly lower than the number estimated by weighing (Table 1) for samples of both 3000 (χ^2^ = 40.81, df = 5, *P* < 0.0001) and 4000 (χ^2^ = 81.87, df = 5, *P* < 0.0001). However, no significant difference was observed for putative batches of 1000 eggs (χ^2^ = 9.575, df = 5, *P* = 0.08). The mean difference between observed and expected number of eggs in larger egg batches was around 10 %.

Comparison between eggs freshly collected from small rearing cages and immediately hatched (control) and eggs collected from the mass-rearing cages through sieves and then dried did not find any difference in hatch rate, meaning that the combined collection and drying processes did not impact the egg hatch rate (fresh: 69.2 ± 3.3 %, dried: 65.6 ±1.6 %: t = 0.741, df = 2, *P* = 0.5).

## Discussion

It is important for the production of insects for SIT to standardize all of the steps in the mass-rearing process from egg collection to pupal production, and so a method to accurately quantify eggs for hatching, quality control etc. is essential. A method has been developed to estimate numbers of *An. arabiensis* eggs involving drying, brushing and weighing. None of these treatments had any negative effect on hatch rate, survival to pupation, or adult fitness, as judged by wing length or longevity.

A highly significant positive correlation was observed between dried, brushed egg numbers and egg weight, a method of quantification validated by comparing the actual number of eggs counted after estimating the number of eggs based on previously measured correlation between egg number and weight. Larger egg batches of 3000 and 4000 eggs were underestimated by weighing, probably due to variation in egg weight after drying. The experiment was carried out with eggs collected on multiple filter papers on which different numbers of eggs were laid. Some filter papers carried thousands of eggs which were overlaid on top of each other; though drying conditions were similar for all batches, such eggs would be less desiccated and so heavier. However, a simple adjustment of 10 % can be made based on the batch size to correct the estimated number of eggs.

Allowing eggs to dry under insectary conditions for 3–5 h before being brushed, weighed and counted, did not impair the hatch rate of the eggs, as was previously observed by Khan et al. [9]. Other studies on *An. quadriannulatus* and *An. albimanus* found that eggs could survive drying and even dry storage [8]. Though Anopheline eggs show some tolerance to desiccation under experimental conditions and can survive the temporary desiccation of an oviposition site in the field [20], they cannot survive prolonged drying [9]. The treatment of eggs for quantification also had no negative effect on any subsequent larval or adult life history traits studied. It is known from previous work by the authors that the robust nature of *Aedes* eggs allows brushing and handling without any decrease in hatch rate [10, 11]. While *Aedes* lay their eggs on a substrate above the water, *An. arabiensis* eggs are usually laid on the water surface and float thanks to two air-filled expansions of the exochorion [20]. It was feared that the brushing might damage the floats and cause the eggs to sink, but if this did happen it did not reduce the hatch rate.

Finally, a more rapid drying technique was tested. In mass-rearing facilities it would not be practical to have hundreds of filter papers drying over a period of 3–5 h. The technique developed by Dame et al. [7] and recently adapted by Khan et al. [9] using a suction device allowed the drying of eggs in half an hour without any loss of egg quality. However, this drying method led to an overestimation of egg number. Because of the air flow, a higher number of eggs collapsed (due to rapid loss of water) than in the eggs subjected to gentle drying (personal observation), though apparently without affecting hatch rate. Therefore, for the same weight, samples from the rapid drying method, because of the presence of collapsed eggs (probably lighter than normal eggs), will have more eggs than samples from the gentle drying method. However, the variation appears to be quite constant and can be taken into consideration for egg number estimation.

When the quantification method was adapted to the mass-rearing cages, the addition of the sieving process prior to egg estimation seems not to have further impacted the egg hatch rate and the method could therefore be used in a mass-rearing setting. However, when the equation linking the egg number and weight was used, the actual observed mean number of eggs was less than the expected number. In mass-rearing cages, when the adults die, they fall onto the water where the females oviposit. Collection by sieving separates eggs from the dead bodies, but some finer debris such as scales, cuticle fragments, partial legs, and thorax, abdomen and wing fragments pass through. So when eggs from mass-rearing cages were weighed, some debris was also included, leading to an underestimation of eggs collected. The difference between observed mean and expected number of eggs was around 10 %, depending on the mortality rates according to the day of egg collection and age of adults. The higher the number of dead mosquitoes in the egging water, the higher the quantity of debris contaminating the eggs. Two options could be envisaged to solve the problem. An additional egg cleaning step previously proposed by Khan et al. [9] could be useful. However, attention should be paid not to damage the eggs since hatch rate is crucial to efficient rearing. Another way could be taking the variation into account by weighing around 110 % of the mass of the desired number of eggs.

## Conclusions

In the present study, an efficient and fast method of mosquito egg collection and quantification has been developed and shown not to impair the quality of eggs. Precise in routine insectary scale rearing, the method was also adapted to quantify eggs coming from mass-rearing cages. The method described here facilitates easy and reproducible seeding, each with the same number of eggs, of the fifty rearing trays held in the rack developed by the IPCL [4]. These results are encouraging as an important step in the development of robust mass-rearing procedures for the SIT against malaria vectors. In further developments, the processes of larval development and pupae collection have also to be standardized. Parameters such as the number of eggs to seed each tray with, the quantity of larval food to give, the timing of pupation, and the management of larvae and pupae during the pupation period will also need to be studied and optimized.
